# Supplementary material for: Soil water depletion patterns in rainfed apple orchards and wheat fields
Source: PeerJ. 2023 Mar 30;11:e15098. doi: 10.7717/peerj.15098 (PMC10066881; doi:10.7717/peerj.15098)
Supplement: Supplemental Information 2 [file peerj-11-15098-s002.pdf]

TCC 超越译文科技咨询 Transcend Envirotech Consulting

eWin Editing Services

LANGUAGE EDITING

CERTIFICATE

This document certifies that the manuscript listed below was edited for proper English language, grammar, punctuations, spelling, and overall style by one or more of the highly qualified native English speaking editors at eWin Editing Services.

Manuscript Title:

Soil water depletion patterns in rain-fed apple orchards and wheat fields

Authors:

Lu Zhang et al.

Date Issued:

January 31, 2023

Certificate Number:

TEC2002530OPDA0131

Disclaimer: This document certifies that the manuscript listed above was edited for proper English language, grammar, punctuation, spelling, and overall style. Neither the research content nor the author's intentions were altered in any way during the editing process. Documents receiving this certification should be English-ready for publication. However, the author has the ability to accept or reject our suggestions and changes. We do not bear responsibility for revisions made to the document after our edit. If you have any questions or concerns about this document or certification, please contact **info@ewinediting.com**.

青岛超越译文科技咨询有限公司 3702820026700

Transcend Envirotech Consulting

Qingdao, China

eWin Editing Services (译文编辑) is an editing brand of Transcend Envirotech Consulting Co., Ltd., which specializes in helping Chinese researchers succeed in publishing internationally. We are committed to providing professional translation, editing and proofreading services in environmental, biological, and agricultural sciences. Our English editing services are provided by a team of native UK editors who have a strong research background.
